# Maternal tobacco exposure and health-related quality of life during pregnancy: a national-based study of pregnant women in China

**DOI:** 10.1186/s12955-021-01785-x

**Published:** 2021-05-20

**Authors:** Weiwei Sun, Xinyu Huang, Huailiang Wu, Casper J. P. Zhang, Zongzhi Yin, Qianqian Fan, Huiyun Wang, Pallavi Jayavanth, Babatunde Akinwunmi, Yanxin Wu, Zilian Wang, Wai-kit Ming

**Affiliations:** 1 Department of Public Health and Preventive Medicine, School of Medicine, Jinan University, Guangzhou, China; 2 International School, Jinan University, Guangzhou, China; 3 School of Public Health, LKS Faculty of Medicine, The University of Hong Kong, Hong Kong, China; 4 Department of Obstetrics and Gynecology, The First Affiliated Hospital of Anhui Medical University, Hefei, China; 5 Department of Pharmacy, Peking Union Medical College Hospital, Beijing, China; 6 School of Pharmacy, JiNing Medical University, Jining, China; 7 Maternal-Fetal Medicine Unit, Department of Obstetrics and Gynecology, Brigham and Womens Hospital, Boston, USA; 8 Center for Genomic Medicine, Massachusetts General Hospital, Boston, USA; 9 Harvard Medical School, Harvard University, Boston, MA USA; 10 Department of Obstetrics and Gynaecology, The First Affiliated Hospital of Sun Yat-Sen University, Guangzhou, China

**Keywords:** First-hand smoke, Second-hand smoke, Third-hand smoke, Pregnancy, Health-related quality of life

## Abstract

**Background:**

With the increase of the number of smokers, tobacco exposure among pregnant women is becoming more and more common. Pregnant women exposed to first-hand smoke and second-hand smoke are susceptible to physiological and psychological health issues has been proved in previous studies. Nevertheless, there are no enough studies focus on the impact of third-hand smoke during pregnancy. This study aimed to assess and compare health-related quality of life for pregnant women with exposure to first-hand smoke, second-hand smoke, third-hand smoke and non-exposure to tobacco in mainland China.

**Methods:**

National-based cross-sectional study is based on a questionnaire survey which collects information including demographics, smoking behaviors and self-evaluation. All questionnaires were delivered and collected from August to September 2019. EuroQol groups visual analog scale and EuroQoL Five-dimension Questionnaire were used to collect data in mainland China.

**Results:**

Totally, 15,682 pregnant women were included in this study, among which non-exposure to smoke were 7564 (48.2%), exposed to first-hand smoke, second-hand smoke and third-hand smoke were 89 (0.6%), 2349 (15.0%), and 5680 (36.2%) respectively. Pregnant women without tobacco exposure had the highest EuroQol groups visual analog scale score (mean value=85.4[SD=14.0]), while those with first-hand smoke had the lowest score (mean value=77.4[SD=22.2]). Among all five dimensions of EuroQoL Five-dimension Questionnaire, there were significant differences of EQ-index among groups with different tobacco exposure in usual activity and anxiety or depression dimensions (*p*<0.001).

**Conclusions:**

Third-hand smoke exposure had close relationship with low health-related quality of life in pregnant women. Moreover, second-hand smoke exposure significantly led more problems on mental dimension of pregnant women.

## Background

Tobacco smoking is a well-known risk factor that can cause series of significant morbidity and mortality worldwide, which accounting for more than 8 million deaths annually in global sphere [1]. However, the number of smokers continued to increase and reached 1.1 billion in the world by 2019 [2, 3]. Asthma, cardiovascular diseases and cancer are the common complications caused by active first-hand smoke (FHS) [4,7]. Except for FHS, second-hand smoke (SHS) and third-hand smoke (THS) are two common ways of passive tobacco exposure [8, 9]. SHS contributes to variable diseases as grave as FHS, and caused an additional deaths of 1.2 million people annually [1]. Although SHS could be avoided by multiple ways for non-smokers, THS is much difficult be avoided because THS indicates residual tobacco smoke and particles deposited on surfaces of subjects and dust which may remain for more than one and half years after smoking [10].

The escalating numbers of smokers cause grave complications in both pregnant women and infants by passive tobacco exposure [11,13]. Studies have stated that although many pregnant women never smoke, they still have great chances to expose to SHS and THS [14], especially smoking from their spouses. A study in Sichuan province, China, has shown that 75.1% of non-smoking pregnant women are victimized due to chronic smoking habits of respective spouses [15]. Previous studies have shown that tobacco exposure can be considered as one of the major risk factors of adverse maternal outcomes such as ectopic pregnancy and spontaneous abortion [16, 17]. Moreover, depression symptoms among pregnant women is also an established glaring fact due to SHS, and under this situation, the probability of stillbirth and fetal congenital malformation increased to 23% and 13% respectively [18]. Prenatal SHS exposure in pregnant women shows variety of adverse effects to infants, such as decline the cognitive functions in infants at 6months [19]. Therefore, it is necessary to evaluate the negative impacts of tobacco exposure and health conditions of pregnant women from multiple dimensions.

World Health Organization (WHO) defines quality of life as an individuals perception of their position in life in the context of the culture and value systems in which they live and in relation to their goals, expectations, standards and concerns [20]. Health-related quality of life (HRQoL) is an indicator shows how well people are able to function and how they feel about physical, mental, and social dimensions of their lives [21]. The idea of concentration on HRQoL is not only beneficial to people, but also has significant meanings to economic and social assessment, and also important to public policy, community programs and legislation [22,24]. Since modern medicine is not only about curing diseases but also more about prevention [23, 25], assessing the HRQoL of pregnant women exposed to tobacco during pregnancy is as important as that of the pregnancy outcomes.

Considering the importance above, this study aimed to investigate the HRQoL of pregnant women exposed to FHS, SHS and THS and compare impacts of different tobacco exposure during pregnancy since previous studies on THS is not enough. In addition, this study also compared HRQoL in pregnant women under different tobacco exposure in different regions, and investigate the five dimensions of HRQoL in pregnant women.

## Materials and methods

### Study design and population

This national-based cross-sectional study was designed to investigate the effects of FHS, SHS and THS on HRQoL in pregnant women from mainland China. Questionnaires used in this study was designed based on the Global Tobacco Surveil-lance System [26], and the EuroQoL Groups five-dimension five-level questionnaire (EQ-5D-5L). EQ-5D-5L consists of the EuroQoL Five-dimension Questionnaire (EQ-5D) and EuroQol groups visual analog scale (EQ-VAS), which is an instrumental questionnaire developed in Europe was used to evaluate the general HRQoL of the people [27, 28]. Previous study has proved that EQ-5D-5L can effectively measure health-related quality of life in pregnant women in the population [29]. The Chinese version of the EQ-5D-5L has been proved to be valid and effective that is commonly used to measure HRQoL [30,32]. Patient-evaluated HRQoL is a comparably objective index to assess a patients health status [33]. Each dimension was measured and compared the HRQoL values of pregnant women among different regions in mainland China.

All participants finished the web-based questionnaire delivered by a national platform (Banmi Online maternity school) from August to September, 2019. Pregnant women from 31 provincial administrative units of mainland China were recruited via the national maternity school platform (Banmi Online maternity school). This online platform provides prenatal educational courses to pregnant women based on the mobile app. Pregnant women who used this national platform were asked to participane this study during August to September, 2019. In total, we collected 16,811 questionnaires from pregnant women aged from 16 to 50years old. Inclusion criteria of this study were as follows: (1) ethnically Chinese women with live pregnancy; (2) used Banmi Online maternity school from August to September 2019. The exclusion criteria were as follows: (1) pregnant women who did not live in mainland China; (2) missing demographic data. As some overseas Chinese pregnant women also used the online platform, those who do not live in mainland China were excluded from this study (n=1114). There were 15 participants to be excluded because of missing data.

According to the standards of Chinese CDC, the study was conducted in seven regions of mainland China: (1) Northeast: Heilongjiang, Jilin, Liaoning; (2) North: Beijing, Tianjin, Hebei, Shanxi, Inner Mongolia; (3) Central: Hubei, Hunan, Henan; (4) East: Shanghai, Shandong, Jiangsu, Anhui, Jiangxi, Zhejiang, Fujian; (5) South: Guangdong, Guangxi, Hainan; (6) Northwest: Shaanxi, Gansu, Ningxia, Xinjiang; (7) Southwest: Chongqing, Sichuan, Guizhou, Yunnan, Tibet.

### Variables and measurement

Demographic data included age, gestational age and addresses (provinces and cities). The primary variables in the study include the smoking states of pregnant women (smokers or non-smokers), and husbands (smoked in proximity, smoked but not in the proximity and not smoked). Key assessments included the EQ-5D index and EQ-VAS values. Husband smoking in proximity indicated SHS exposure because pregnant women were directly exposed to tobacco. Husband smoked but not in the proximity indicated THS exposure. Based on previous studies [10, 34], although pregnant woman were not exposed to SHS directly, they were also exposed to residual tobacco smoke and particles deposited on clothes and other subjects. For those pregnant women who were non-smokers and their husbands did not smoke were categorized as non-tobacco exposure.

The EQ-5D-5L system is a measurement that includes five dimensions: (1) mobility; (2) self-care; (3) usual activities; (4) pain or discomfort; (5) anxiety or depression. Each question corresponds to five levels: none; slight; moderate; severe; and extreme severe or unable. Each level in each question is represented by an integer value from 1 to 5 [35, 36]. Through the EQ-5D indicator value calculator, the values of different levels for each question are arranged and can be calculated as a single EQ-5D indicator value (such as 12,121) to generate the final HRQoL value. In this manner, 1 indicating the best health state while 0 represents death [28, 29, 31]. Range of EQ-index value is in the interval of0.224 to 0, and these negative values represent their overall health states (both physical and mental state) are worse than death [31].

EQ-VAS is a self-assessment of respondents' health status. It is presented as a vertical line, dividing from 100 (the imaginable best state of health) to 0 (the imaginable worst state of health). Respondents were asked to draw a line on this scale based on their views on their health status, filling the score in the blank space next to it [37].

### Statistical methods

Data analysis was performed by using Statistical Product and Service Solutions (SPSS) 16.0 for Mac and 25.0 for Win. Normally distributed continuous variables were analyzed by independent sample analysis, and were described using the means standard deviations (SDs). The categorical variables were described using counts and percentages. The dependent variables were the EQ-index and EQ-VAS in a skewed distribution; therefore, we used a non-parametric approach to analyze the data.

For the different dimensions in EQ-5D-5L questionnaires, One-Way Analysis of Variance (ANOVA) and non-parametric tests were used to calculated the data. Multiple comparison analysis was also used to compare the difference between groups of exposure. All tests were two-sided, and *p* value of 0.05 was considered as statistically significant.

## Results

As shown in Table 1, the samples (15,682 in total) were in the average age of 28.6 (4.7) and in average gestational age of 21.0 (9.2), including 7564 pregnant women without tobacco exposure, 2349 pregnant women with SHS exposure, and 5680 pregnant women exposed to THS. EQ-index and EQ-VAS of pregnant women in different groups were reported in Table 1. There was significant difference of EQ-VAS scores among pregnant women with different tobacco exposure (*p* value<0.001).

**Table 1:** The demographic and health-related quality of life of pregnant women with different types of exposure to tobacco

Total participants (*N*=15,682)	Non-exposure (*N*=7564)	FHS (*N*=89)	SHS (*N*=2349)	THS (*N*=5680)	*p* value
Age (SD)	28.6 (4.7)	26.2 (5.1)	27.5 (5.2)	28.6 (4.7)	<0.001*
Gestational age (SD)	21.0 (9.2)	21.8 (9.0)	21.5 (9.2)	21.1 (9.0)	0.82
EQ-index (SD)	0.804 (0.13)	0.808 (0.14)	0.796 (0.13)	0.807 (0.13)	0.07
EQ-VAS (SD)	85.4 (14.0)	77.4 (22.2)	80.6 (17.6)	84.5 (14.9)	<0.001*

^*^p value<0.05 indicates the statistical difference

The EQ-VAS was in a skewed distribution, and not in equal variance (<0.001). Therefore, we used Tahmane's T2 method to compare EQ-VAS of pregnant women between different tobacco exposure groups in Table 2. There were obvious differences in EQ-VAS between non-tobacco exposure and tobacco exposure groups in respect of FHS, SHS, and THS (*p*=0.007,<0.001, 0.001 respectively). To be specific, the average score of EQ-VAS for pregnant women with THS exposure was significantly higher than those with FHS and SHS exposed pregnant women (*p*=0.024 and<0.001 respectively). But there was no significant difference in EQ-VAS between FHS pregnant women and SHS pregnant women (*p*=0.729).

**Table 2:** Multiple comparisons of EQ-VAS (Tamhanes T2 method) for pregnant women in different types of tobacco exposure

Types of tobacco exposure	EQ-VAS	Comparison group	Mean EQ-VAS score (SD)	Mean difference	Standard error	*p* value	95% confidence interval	
							Upper bound	Lower bound
Non-exposure	85.4 (14.0)	FHS	77.4 (22.2)	0.81	2.39	0.007*	14.44	1.59
		SHS	80.6 (17.6)	4.88	0.40	<0.001*	5.92	3.83
		THS	84.5 (14.9)	0.95	0.26	0.001*	1.62	0.28
FHS	77.4 (22.2)	Non-exposure	85.4 (14.0)	0.81	2.39	0.007*	1.59	14.44
		SHS	80.6 (17.6)	3.14	2.41	0.729	3.34	9.61
		THS	84.5 (14.9)	7.06	2.39	0.024*	0.63	13.49
SHS	80.6 (17.6)	Non-exposure	85.4 (14.0)	4.88	0.40	<0.001*	3.83	5.92
		FHS	77.4 (22.2)	3.14	2.41	0.729	9.61	3.34
		THS	84.5 (14.9)	3.92	0.41	<0.001*	2.84	5.01
THS	84.5 (14.9)	Non-exposure	85.4 (14.0)	0.95	0.26	0.001*	0.28	1.62
		FHS	77.4 (22.2)	7.06	2.39	0.024*	13.49	0.63
		SHS	80.6 (17.6)	3.92	0.41	<0.001*	5.01	2.84

^*^P value<0.05 indicates the statistical difference

In Table 3, we displayed the different numbers in different levels of EQ-5D dimensions for pregnant women under different types of tobacco exposure, to evaluate the impact on SHS and THS on the different aspects. Among all five dimensions, no matter which types of tobacco exposure, more than half of pregnant women had health problems (value 25) on pain or discomfort and anxiety or depression dimensions. There were significant differences of EQ-index scores in usual activity and anxiety or depression dimensions between pregnant women in different groups (both *p*<0.001).

**Table 3:** Levels of EQ-5D dimensions for pregnant women exposed to different types of tobacco

EQ-5D dimension	Non-exposure	FHS exposure	SHS exposure	THS exposure	*p* value
	*N*=7564	*N*=89	*N*=2349	*N*=5680	
*Mobility*					0.693
1	5832 (77.1%)	70 (77.8%)	1833 (78.0%)	4426 (77.9%)	
2	1460 (19.3%)	17 (18.9%)	423 (18.0%)	1057 (18.6%)	
3	217 (2.9%)	2 (2.2%)	77 (3.3%)	158 (2.8%)	
4	27 (0.4%)	0 (0.0%)	9 (0.4%)	20 (0.4%)	
5	28 (0.4%)	0 (0.0%)	7 (0.3%)	19 (0.3%)	
*Self-care*					0.067
1	7077 (93.6%)	81 (90.0%)	2223 (94.6%)	5376 (94.6%)	
2	446 (5.9%)	4 (4.4%)	111 (4.7%)	277 (4.9%)	
3	28 (0.4%)	2 (2.2%)	11 (0.5%)	20 (0.4%)	
4	4 (0.1%)	1 (1.1%)	3 (0.1%)	4 (0.1%)	
5	9 (0.1%)	1 (1.1%)	1 (0.0%)	3 (0.1%)	
*Usual activity*					<0.001*
1	5925 (78.3%)	74 (82.2%)	1904 (81.1%)	4609 (81.1%)	
2	1,472 (19.5%)	10 (11.1%)	405 (17.2%)	960 (16.9%)	
3	121 (1.6%)	2 (2.2%)	33 (1.4%)	87 (1.5%)	
4	17 (0.2%)	1 (1.1%)	4 (0.2%)	7 (0.1%)	
5	29 (0.4%)	2 (2.2%)	3 (0.1%)	17 (0.3%)	
*Pain or discomfort*					0.218
1	3299 (43.6%)	44 (48.9%)	988 (42.1%)	2523 (44.4%)	
2	3291 (51.8%)	39 (43.8%)	1243 (52.9%)	2908 (51.2%)	
3	295 (3.9%)	5 (5.6%)	107 (4.6%)	218 (3.8%)	
4	39 (0.5%)	1 (1.1%)	8 (0.3%)	27 (0.5%)	
5	10 (0.1%)	0 (0.0%)	3 (0.1%)	4 (0.1%)	
*Anxiety or depression*					<0.001*
1	3773 (49.9%)	36 (40.0%)	1000 (42.6%)	2824 (49.7%)	
2	3380 (44.7%)	40 (44.4%)	1123 (47.8%)	2549 (44.9%)	
3	339 (4.2%)	12 (13.3%)	158 (6.7%)	256 (4.5%)	
4	50 (0.7%)	0 (0.0%)	49 (2.1%)	41 (0.7%)	
5	22 (0.3%)	1 (1.1%)	19 (0.8%)	10 (0.2%)	

^*^p value<0.05 indicates the statistical difference

**Fig. 1:**
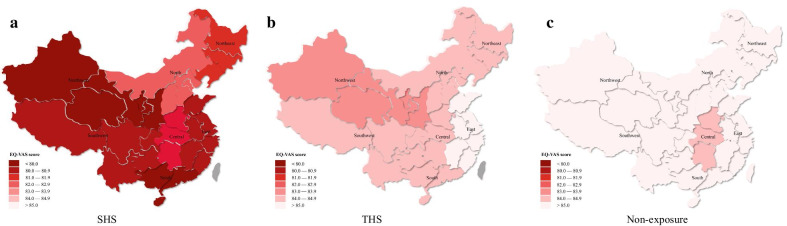
EQ-VAS for pregnant women under different tobacco exposure condition in different regions of mainland China

For Fig.1, we depicted the EQ-VAS for pregnant women under different tobacco exposure condition in different regions of mainland China. Pregnant women without tobacco exposure had the highest EQ-VAS value, while pregnant women exposed to SHS had the lowest EQ-VAS value. Pregnant women lived in the northwest region showed obvious lower EQ-VAS lower with the exposure of SHS and THS which indicated a lower level of HRQoL. Besides, pregnant women lived in north region with SHS and THS exposure showed relative higher EQ-VAS score than other regions.

## Discussion

This study explored and compared the HRQoL of pregnant women exposed to FHS, SHS and THS in mainland China. Overall, it showed that tobacco exposure during pregnancy can lead to lower level of HRQoL regardless of the types of exposure (FHS, SHS and THS). Furthermore, our results emphasized that FHS and SHS could cause more severe effects on HRQoL of pregnant women than THS. Also, tobacco exposure during pregnancy has been proved to be a risk factor for HRQoL of pregnant women in this study. This finding is consistent with previous studies in other countries [38,41]. The important health issues and the adverse effects caused by direct maternal tobacco smoking and passive SHS exposure will significantly harm not only pregnant women but also their fetuses or newborns [42,46]. However, there was no previous study examined and compared the impacts of pregnant women exposed to FHS, SHS and THS. This study is the pioneer project to provide empirical evidence on the adverse effect of THS in addition to FHS and SHS. We found that THS had close relationship with lower HRQoL of pregnant women. Although others didnt smoke in front of pregnant women, THS exposure still remained due to residues. Even if people with lower HRQoL might be asymptomatic in the clinic, previous study has found that HRQoL has close association with the state of health and clinical outcomes [47].

As mentioned above, it was found that THS had certain adverse impact on HRQoL of pregnant women, but FHS and SHS had more severely negative impacts. This may be explained by the facts that the mechanism of THS was residual tobacco smoke gases and particles settled on surfaces, and they would enter the human body through dermal absorption and ingestion [48, 49]. Through this, the epithelia and mucosa of the respiratory tract could act as protective barriers to prevent the harmful materials and cause a relatively lower level of nicotine dose in human body [50]. Even so, all these three types of tobacco exposure can lead to lower level of HRQoL of pregnant women. Compared to the research findings of other previous studies, we found that pregnant women exposed to FHS and SHS have a similar, and sometimes even much lower HRQoL than those suffered from physiological diseases, such as gestational diabetes mellitus and uterine fibroid [29, 51]. Although electronic cigarettes has been considered as a safer way than smoking tobacco among people recent years [52], recent studies have stated that e-cigarette products still can cause varying degree of lung damage and chronic respiratory symptoms and both DNA strand breaks and cell death [53,55]; Thus, the usage of e-cigarettes should also be avoided. Overall, it is strongly recommended that pregnant women and their spouses should quit smoking during pregnancy to avoid the massively negative effects of any types of tobacco exposure (FHS, SHS, THS and e-cigarette).

In addition, Fig.1 revealed that northwest region had relatively lower level of HRQoL under any type of tobacco exposure. This might be related to economic conditions and healthy awareness of pregnant women and their relatives. Previous study has shown that economic conditions are associated with HRQoL and northwest region had a relatively backward economic condition among total seven regions in mainland China [22, 56].

Moreover, this study further analyzed and compared the five dimensions in the EQ-5D-5L scale. It was found that in usual activity and anxiety or depression, different tobacco exposure showed significant differences, especially on anxiety or depression dimension. Pregnant women exposed to SHS had a higher anxiety or depression rate (57.4%) than those exposed to THS (50.3%), which was strongly related to psychological health problems. Preceding findings have shown that exposure to SHS can lead to mentally stressful living environments, while chronic stress or other comorbidities may increase the risk of prevalence of mental disorders [57, 58], indicated a strong correlation between exposure to SHS and negative health effects (such as cancer, respiratory diseases), and all these diseases may lead to depression through direct and indirect multi-step processes. Besides, there was a strong evidence that major depression had close association with SHS exposure [59]. Animal studies showed that SHS adversely affects the dopaminergic system [60]. With long-term exposure to SHS, the levels of dopamine and -aminobutyric acid (GABA) are reduced, which is also associated with an increased risk of depression [61].

In summary, exposure to tobacco has certain negative impacts on the HRQoL of pregnant women even when their spouses did not smoke in the proximity of them. A better HRQoL is more conducive to the health of both pregnant women and fetuses. These findings can help to evaluate the negative impacts of different types of tobacco exposure during pregnancy and provide more clinical evidences on the implementation of pregnant tobacco-control policies. According to these, we call on higher level of healthy education during pregnancy for pregnant women themselves and their spouses and other household relatives, because they may do not have enough understanding of HRQoL and lack awareness about harmful effects of tobacco exposure. Moreover, spouses and household relatives should avoid smoking in front of pregnant women even if smoking is unavoidable. Due to the evidence that THS could also affect the HRQoL of pregnant women, future clinicians and scientists can pay more attention to the study of THS, to ensure overall better pregnancy outcomes.

## Strengths and limitations

The main strength of this study is to focus on HRQoL in pregnant women exposed to different types of exposure to tobacco smoke in China, especially the comparison between SHS and THS. In addition, the large sample size from different regions in mainland China contributes to the good understanding and comparisons of HRQoL of pregnant women in different areas. The major limitation is that the EQ-5D and EQ-VAS are relatively subjective measurements of pregnant womens HRQoL. Thus, the self-reported bias may be the main bias in this study. Less data of pregnant women exposed to FHS was another limitation, this might be explained by that a high level of prenatal education in China, and most pregnant women do not actively smoke. Our aim was to understand the impact of the husband on pregnant women during pregnancy, so as to give pregnant women and their husbands some clinical recommendation. However, potential SHS and THS exposures of pregnant women coming from different sources rather than their husbands are not considered, such as tobacco exposure from the place where pregnant women work or perform other daily activities. And we will conduct follow-up studies to explore the impact of tobacco exposures from different sources on pregnant women. Other socio-demographic factors such as economic situation may also affect our results. Figure1 was based on pregnant women's geographical location, and the results showed HRQoL of pregnant women in the northwest region, which had relatively backward economic conditions [22, 56], was slightly lower than in other regions. However, we did not have the individual level of socio-demographic factors which was a limitation to this aspect.

## Conclusions

Pregnant women exposed to tobacco gases andparticles had significantly lower HRQoL regardless of types of tobacco exposure (FHS, SHS and THS). FHS and SHS exposure could cause more health problems on pregnant womens mental health than THS exposure. Therefore, our study advocates that pregnant women and their spouses should quit smoking during pregnancy. If tobacco exposure cant be avoided in some special situation, refraining from smoking in front of pregnant women would be a better choice. Besides, the government should strengthen the prenatal education for pregnant women and their spouses to introduce the specific hazards of both active and passive smoking, increasing their awareness to protect the pregnant women from the harms of tobacco.

## Data Availability

The datasets used and analyzed during the current study are available from the corresponding author on reasonable request.

## Abbreviations

FHS: First-hand smoke
SHS: Second-hand smoke
THS: Third-hand smoke
HRQoL: Health-related quality of life
